# A Rare Case of Recurrent Intra-abdominal Desmoid-Type Fibromatosis

**DOI:** 10.7759/cureus.69049

**Published:** 2024-09-10

**Authors:** Shuhaini Musa, Jyotsna Kakarla, Sangara Narayanasamy, Ali Yasen Mohamedahmed, Stelios A Vakis

**Affiliations:** 1 General Surgery, Queen's Hospital Burton, University Hospitals of Derby and Burton NHS Foundation Trust, Burton-on-Trent, GBR; 2 General and Colorectal Surgery, Queen's Hospital Burton, University Hospitals of Derby and Burton NHS Foundation Trust, Burton-on-Trent, GBR

**Keywords:** desmoid tumours, desmoid-type fibromatosis, fibroblasts, intra-abdominal mass, spindle-shaped cells

## Abstract

Desmoid-type fibromatosis is an uncommon fibroblastic or myofibroblastic tumour arising in deep soft tissues with no metastatic potential. This case report presents a 78-year-old male patient with an incidental finding of desmoid-type fibromatosis of the abdomen with recurrence within two years and required surgical interventions. Primarily, a computed tomography (CT) of the abdomen and pelvis showed an incidental finding of a large soft tissue mass in the right iliac fossa mesentery measuring 11 by 8.5 cm. The patient underwent a successful elective exploratory laparotomy and resection of the mass along with a small bowel. A final pathology revealed the mass to be a primary desmoid of the small bowel. Despite clear resection margins, the patient developed recurrence after 17 months, which was treated with surgical resection. His post-operative course was uneventful. The patient’s clinical presentation, management, and diagnosis are discussed in this case report.

## Introduction

Desmoid-type fibromatosis is a rare and locally aggressive soft tissue tumour with a tendency towards local recurrence and no ability to metastasise [1]. The reported incidence of desmoid tumours is about two to four per million population, representing 0.03% of all neoplasms [2]. It is commonly occurred as sporadic; however, 5-10% of the cases are related to familial adenomatous polyposis (FAP). Patients with FAP have a higher risk of developing desmoid-type fibromatosis, and it will commonly occur intraabdominally within the mesentery or in the abdominal wall compared to the general population or sporadic cases [1]. Desmoid-type fibromatosis is also associated with pregnancy and the use of oral contraception, although its pathogenesis remains unclear.

Clinical presentations can be varied depending on the location of the tumour. Patients can remain asymptomatic or present with painless mass until the tumour size is large enough to affect the adjacent neurovascular structures or other internal organs, causing complications such as bowel obstruction or hydronephrosis [3]. The computed tomography (CT) scan is the most common imaging test to confirm the diagnosis, especially for intra-abdominal mass. The magnetic resonance imaging (MRI) scan is the preferred imaging test for abdominal wall or extra-abdominal mass. Surgical resection is the mainstay treatment approach for desmoid tumours. Still, as it is associated with post-operative complications, morbidity, and mortality, a stepwise approach has been described in selected cases in asymptomatic patients, with ‘wait and see’ or active surveillance as the initial plan [1].

## Case presentation

**Figure 1:**
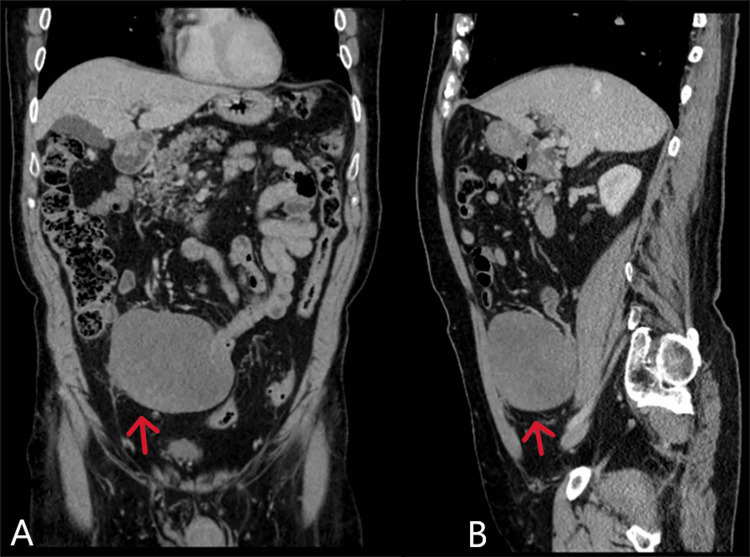
CT abdomen and pelvis (coronal section labelled A and sagittal section labelled B) demonstrating the mass in the right iliac fossa (red arrows).

A 78-year-old male, with a medical history including psoriasis and myositis, has been undergoing gastroenterology monitoring because of an asymptomatic abnormal liver function test (LFT). Alanine transaminase (ALT) was 220 units per litre (U/L) (normal range: 7-55 U/L), alkaline phosphatase (ALP) was 185 U/L (normal range: 40-129 U/L), and the rest of the LFT was unremarkable. An interval surveillance CT scan in January 2022 did not detect any abnormalities related to the liver or gallbladder. However, an incidental discovery of a sizable soft tissue mass measuring 11 by 8.5 cm in the right iliac fossa mesentery was made, prompting radiologists to suggest potential diagnoses such as a desmoid tumour, carcinoid, or a gastrointestinal stromal tumour (GIST) (Figure 1).

**Figure 2:**
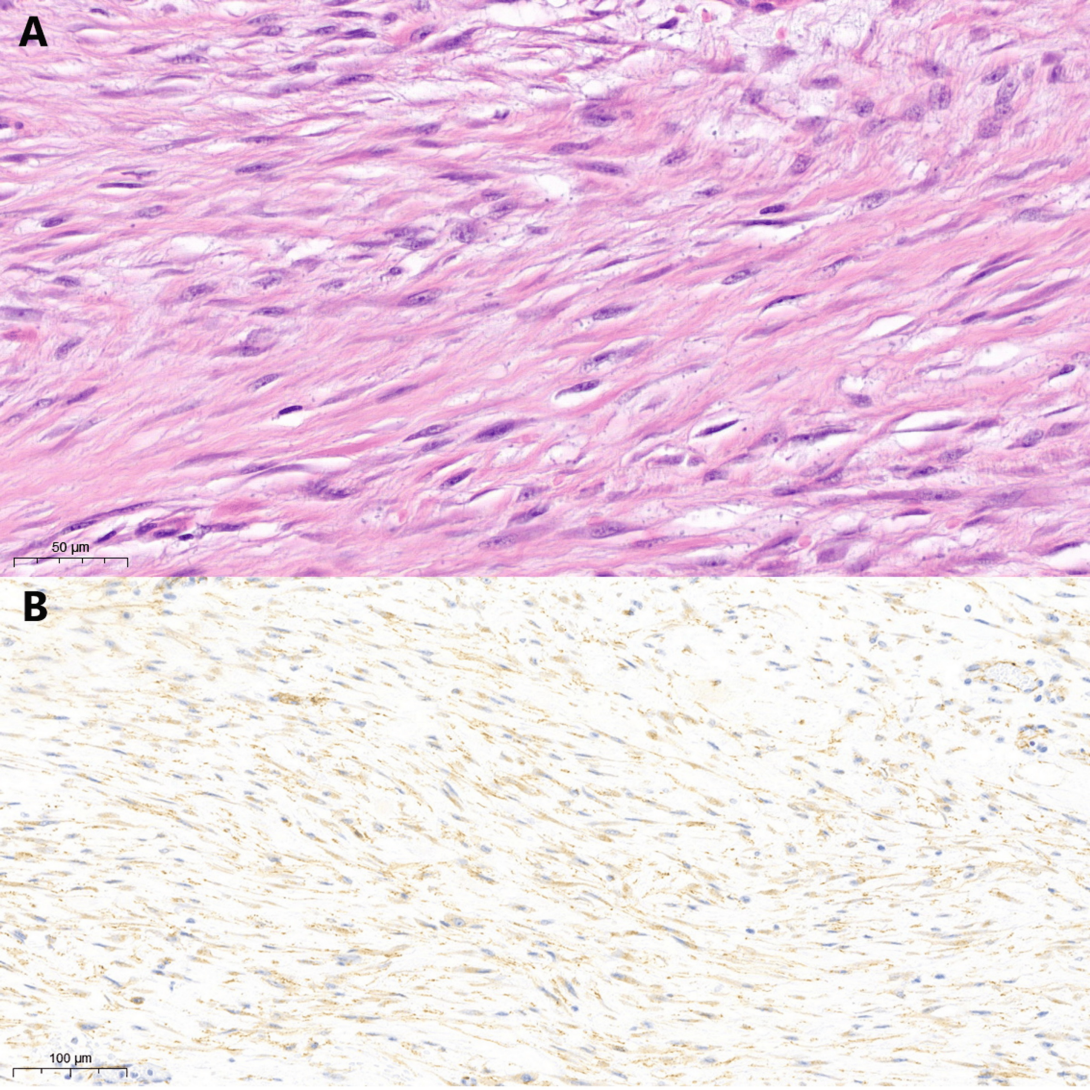
Histopathology slide images of the resected mass. The top slide image (labelled A) is H&E-stained and shows sweeping fascicles of uniform spindle cells (magnification: 40×). The bottom slide (labelled B)image shows positive results with immunohistochemistry for beta-catenin (magnification: 20×). H&E: hematoxylin and eosin

Upon clinical examination, the mass was readily palpable in the midline extending to the right lower abdomen. In March 2022, the patient underwent laparotomy and small bowel resection to address the large intra-abdominal mass. Intraoperative findings indicated a substantial mass arising from the small bowel, extending into the small bowel mesentery with multiple vessel feedings, and no apparent lymphadenopathy. The mass was excised with clear margins, and the histopathology results revealed desmoid-type fibromatosis (Figure 2).

**Figure 3:**
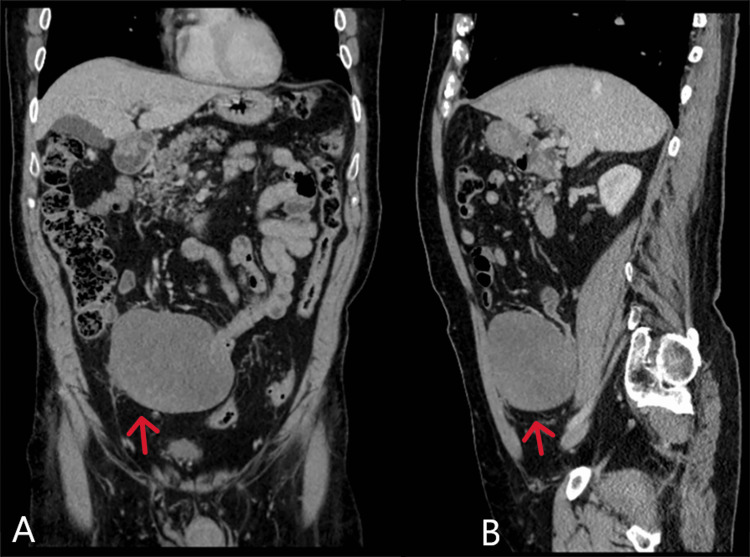
CT abdomen and pelvis (coronal section labelled A and sagittal section labelled B) demonstrating the mass in the left iliac fossa (red arrows).

The patient remained under surgical follow-up because of the large size and potential for local recurrence. A subsequent CT scan in February 2023 showed no tumour recurrence, and the patient remained asymptomatic, with clinical examination yielding no abnormalities. However, in August 2023, the patient presented with a new abdominal mass resembling the previous one. Upon examination, the mass was mobile and palpable to the left side of the abdomen. CT imaging confirmed the presence of a new 9.8 by 10 cm mass lesion in the mesentery on the left side (Figure 3).

In December 2023, the patient underwent laparotomy and resection of the small bowel mesenteric tumour, revealing that the new tumour was unrelated to the original resection site. The histopathology again confirmed a desmoid-type fibromatosis tumour. The patient recovered without complications and continues to undergo surgical follow-up with repeat CT scans for assessment.

## Discussion

The histologic hallmark of desmoid tumours is spindle cells and fibroblasts in the background of collagen stoma [2]. Desmoid-type fibromatosis is a rare and locally aggressive fibroblastic tumour characterised by infiltrative growth and a tendency to recur. In most cases, it is a deeply seated, painless, or minimally painful mass with a history of slow progress. While the lesions may affect skeletal muscle, they often develop in the anterior abdominal wall and shoulder girdle [3,4]. Peripheral desmoid tumours are stable, smooth, and mobile. These tumours commonly adhere to nearby structures without evident infiltration of the overlying skin. Intra-abdominal desmoid tumours typically manifest as sizable neoplasms, often remaining asymptomatic until their growth can cause compression of visceral organs. Initial clinical presentations may include intestinal, neural, vascular, or ureteric obstruction symptoms [2,5,6]. Pain, perforation of hollow organs, functional limitations, fistula formation, and other potentially life-threatening complications arise because of local tissue infiltration [2,3]. However, this reported case was asymptomatic, and a desmoid tumour was found upon incidental finding.

Literature showed an overall recurrence rate of 39.3% and 50% in patients who had a previous surgical resection with a relapse period ranging from three to 26 months [4,7]. Risk factors for recurrence after surgical resection include younger age, extra-abdominal tumour, invasion of major vessels and nerves and the quality of surgical margins [4]. This is reflected in this reported case, where the patient had a relapse within 17 months post-surgical resection. However, this patient is neither in the young age group nor involves an extra-abdominal tumour that could be associated with a risk of recurrence.

Previous case reports described various disease progressions for desmoid-type fibromatosis (DF). Hapgood et al. reported a case of a 47-year-old female who underwent surgical resection twice within four years for recurrent DF in small bowel mesentery [8]. However, there was no recurrence during two years of follow-up in two case reports of DF, intra-abdominal and chest wall DF [9,10]. Comparing these case reports to our case, their patients’ age ranges between 40 and 60 years old, while the current reported patient is slightly in the older age category. Moreover, none of these patients have risk factors associated with desmoid-type fibromatosis.

Traditionally, surgical resection has been the treatment of choice, intending to achieve negative margins, but it is also known to be associated with high morbidity and mortality risks [11]. In several case reports, surgical resection was planned after the imaging tests revealed the mass or desmoid tumour causing the symptoms or the clinical presentation [8-12]. A similar approach is reflected in our case, after which finding the incidental mass on the CT scan, the plan for surgery was initiated. However, in recent years, it has been recommended that all cases have a period of active surveillance so patients can avoid surgical procedures [11]. Studies reported that patients with asymptomatic DF may be initially managed with active surveillance, as a notable percentage of these tumours remain stable after an extended period, even without treatment [1]. In one study involving 83 desmoid fibromatosis patients placed on active surveillance, half had a five-year progression-free survival, and the median time to progression was 14 months [13]. As most cases reach a stabilisation period and some show regression of the tumour, this period of ‘watch and wait’ allows the clinicians to observe the disease progression and make a better management plan [11].

The National Comprehensive Cancer Network (NCCN) recommends follow-up with imaging every three to six months after surgical resection of DF for the first three years and six to twelve months afterwards [14]. However, treatment should be offered to those who decline observation and if the tumour rapidly progresses, causing significant symptoms or complications, requiring active interventions [13]. Other than surgical resection, systematic treatments such as non-steroidal anti-inflammatory drugs (NSAIDs), anti-hormonal therapies, tyrosine kinase inhibitors (TKI), and conventional low-dose chemotherapeutic regimens can be considered [11]. In view that these agents have a high toxicity profile, patients should be monitored closely for any adverse effects. Radiotherapy is another modality of treatment for desmoid tumours, especially in the case of unresectable or recurrent DF, as well as in patients who are at high risk for surgery. A recent meta-analysis reported improved local control rates in the combination of surgery and radiotherapy, whereas surgery alone has a consistently high local recurrence rate [15].

## Conclusions

Desmoid-type fibromatosis could be challenging for surgeons because of its rare incidence and limited available management guidelines. Moreover, the clinical presentations can vary from asymptomatic to significant complications as the tumour affects the adjacent organs. Imaging tests are crucial initial steps of the diagnostic approach, followed by biopsy for histopathology assessment. Regarding management, active surveillance is recommended for asymptomatic desmoid tumours. However, surgical resection aiming for R0 resection can be offered for complicated symptomatic patients. In this reported case, this patient was managed with surgical interventions and is on long-term clinical and radiological surveillance.
